# Illumina-based transcriptomic analysis of the fast-growing leguminous tree *Acacia crassicarpa*: functional gene annotation and identification of novel SSR-markers

**DOI:** 10.3389/fpls.2024.1339958

**Published:** 2024-08-29

**Authors:** Shougo Ishio, Kazutaka Kusunoki, Michiko Nemoto, Tadayoshi Kanao, Takashi Tamura

**Affiliations:** ^1^ Tsukuba Research Institute, Sumitomo Forestry Co. Ltd., Tsukuba, Japan; ^2^ Graduate School of Environment, Life, Natural Science and Technology, Okayama University, Okayama, Japan; ^3^ Institute of Global Human Resource Development, Okayama University, Okayama, Japan

**Keywords:** *Acacia crassicarpa*, illumina sequencing, polymorphism, auxin response factor, lignin

## Abstract

*Acacia crassicarpa* is a fast-growing leguminous tree that is widely cultivated in tropical areas such as Indonesia, Malaysia, Australia, and southern China. This tree has versatile utility in timber, furniture, and pulp production. Illumina sequencing of *A. crassicarpa* was conducted, and the raw data of 124,410,892 reads were filtered and assembled *de novo* into 93,317 unigenes, with a total of 84,411,793 bases. Blast2GO annotation, Benchmark Universal Single-Copy Ortholog evaluation, and GO-term classification produced a catalogue of unigenes for studying primary metabolism, phytohormone signaling, and transcription factors. Massive transcriptomic analysis has identified microsatellites composed of simple sequence repeat (SSR) loci representing di-, tri-, and tetranucleotide repeat units in the predicted open reading frames. Polymorphism was induced by PCR amplification of microsatellite loci located in several genes encoding auxin response factors and other transcription factors, which successfully distinguished 16 local trees of *A. crassicarpa* tested, representing potentially exploitable molecular markers for efficient tree breeding for plantation and biomass exploitation.

## 1 Introduction

Acacia is a fast-growing leguminous tree that can be harvested within a relatively short period (several years). The genetic traits for such rapid carbon fixation allow for a rapid cycle of harvesting and reforestation of tree plantations. Because of their ability to thrive in degraded soils, even under drought conditions (Pan and You, 1994), these trees are planted in large areas of Southeast Asia and Southern China (Midgley and Turnbull, 2003). Acacia crassicarpa is a preferred leguminous tree with valuable properties, such as high wood density, excellent biomass yield, low moisture content, and high combustion heat. Because of the intrinsic ability of trees to store abundant plant nutrients K+, Ca2+, and Mg2+ from the soil, their ash can become highly alkaline with a pH greater than 12 (Yusiharni and Gilkes, 2012). Large-scale burning of wood produces residual alkaline ash, which is highly sticky and causes significant mechanical damage to movable floors in biomass power plants (Fuller et al., 2019; Hallgren et al., 1999). These properties of wood species present breeding challenges that must be solved, and accurate genomic information and molecular breeding techniques are required to address these challenges.

Because traditional breeding of tree species has intrinsic limitations owing to their slow growth and naturally long lifecycles, it is highly challenging to select improved varieties that have the desired genetic traits. Molecular breeding of commercial plants requires several basic resources such as genome sequence information and annotation of genes responsible for target traits. A. crassicarpa has a relatively large genome of 1,350 Mbp (Mukherjee and Sharma, 1995), which is approximately double that of the model species Acacia mangium, at 635 Mbp (Blakesley et al., 2002). Genetic modification of A. crassicarpa is difficult without precise sequence data, such as those obtained from expressed sequence tag (EST) libraries. Efforts have been made to construct transcriptomic databases and microsatellite markers for Acacia tree species (McKinnon et al., 2018), and a few pioneering studies have identified a limited number of ESTs. For example, in A. mangium, 8,963 of 10,752 clones of a cDNA library were constructed via conventional molecular cloning and sequencing (Suzuki et al., 2011).

Massively parallel sequencing of cDNA libraries (RNA-Seq) has become a valuable tool for genome analysis, and is rapidly replacing ESTs for structural and functional genome analysis in plants (Pashley et al., 2006). The illumina-based technology permits the investigation of spliced transcripts, including alternative splicing, leading to the large-scale discovery of novel transcripts and the identification of gene boundaries at single-nucleotide resolution.

Plant somatic cells can undergo dedifferentiation to give rise to a pluripotent cell mass called a callus, which can potentially regenerate new organs or the whole plant (Sugimoto et al., 2010). Proliferative plant somatic cells also provide the opportunity to introduce foreign genes to alter their genetic traits. After genetic alteration, it is necessary to induce differentiation of callus cells into an adventitious shoot and then rooting to establish a plant body. The cellular reprogramming process involved several genetic perturbations induced by physical injury of explant and supplementation of auxin and cytokinin. Elucidation of the gene regulatory mechanism, involving several transcription factors, signaling pathway components, epigenetic alteration of chromatin, and activation of biosynthetic pathways for growth and propagation, may give us clue how to reverse the transformed callus back to the differentiated plant body.

In this study, de novo parallel sequencing was performed to characterize the transcriptome of the pluripotent state of A. crassicarpa. To our knowledge, this is the first illumination-based transcriptomic analysis of A. crassicarpa. The cultured cells provided sufficient RNA without severe mechanical shearing or chemical damage. The annotation of gene ontology (GO) terms was established with reference to the model plant Arabidopsis thaliana and the related tree Prosopis alba. Illumina sequencing data also enabled the identification of microsatellites containing simple sequence repeats (SSR). Unigenes expressing a high degree of polymorphism may reveal hidden links between genotypic diversity and physiological function, which may lead to segregation of subspecies to obtain desirable wood properties.

## 2 Materials and methods

### 2.1 Plant material and RNA isolation

Callus tissues were obtained from *A. crassicarpa* seedlings. The seeds were purchased from Australian Tree Seed Center (ATSC) of the Commonwealth Scientific and Industrial Research Organization. Acacia seeds were soaked in concentrated sulfuric acid overnight to burn off the surface wax, and the seed husk was then removed by washing with tap water. The seeds were sterilized with bleach (0.05% chlorine), transferred onto solid 1/2MS medium containing 0.5 ppm TDZ and 1.0 ppm IAA, and cultured for 30 days at 32°C until callus development.

For transcriptome sequencing, 0.1 g of tissue pieces were manually homogenized with a pestle and vortexed for 2 min using super beads. The total RNA was fractionated using an automated RNA extraction system (Maxwell RSC48, Maxwell Plant RNA Kit; Promega, Tokyo, Japan). RNA integrity was determined using a NanoDrop spectrophotometer and Agilent Bioanalyzer (Agilent Technologies Japan, Ltd.). For SSR analysis, 16 individual tree of *A. crassicarpa* were harvested from the Seed Production Area, Conn, Australia, were purchased from the ATSC.

### 2.2 Complementary DNA library construction and sequencing

A cDNA library was constructed from the mRNA using the Illumina TruSeq RNA Sample Preparation Kit (Illumina Inc. CA, USA). The cDNA isolated using AMPure XP beads was subjected to an end-repair process that converted the overhanging nucleotide ends into blunt ends. The blunt-end cDNAs were then incubated with adenylate to attach to the 3′ end; thus, adaptor DNA bearing a 5′-T overhang could capture the cDNA library. DNA was amplified by PCR and the PCR primer cocktail was annealed onto adaptor sequences. The Illumina next-generation sequencing protocol uses a sequencing-by-synthesis approach with four proprietary nucleotides that possess a reversible fluorophore and terminating properties. A series of images, each representing a single-base extension at a specific cluster, were recorded as the sequencing cycle was repeated at specific clusters on the flow cell surface. Raw sequence reads were deposited in the DNA Data Bank of Japan and Sequence Read Archive database (Experiment DRX208625; Analysis Run DRR218312).

### 2.3 Data analysis and estimation of abundance

Raw sequence data were checked for quality using the Fast QC algorithm (https://www.bioinformatics.babraham.ac.uk/projects/fastqc/). The trimmomatic algorithm was then employed to trim the sequence data and to detect and remove adapter sequences (Bolger et al., 2014). This process performs in silico normalization of the total reads to reduce the number of reads that are subject to de novo assembly, thus improving the runtime of the assembly required for the following Trinity algorithm. A large volume of raw data to be analyzed by paired-end sequencing was reconstructed de novo. The relative abundance of transcripts was estimated and quantified using the RSEM 1.2.15 machine (https://www.encodeproject.org/software/rsem/) in the Trinity program (Grabherr et al., 2011).

### 2.4 Sequencing analysis and annotation

For sequence analysis, all unigenes were initially aligned using BLASTx (e-value < 10^-5^) to protein databases such as NCBI non-redundant proteins (nr) (Altschul et al., 1990), Swiss-Prot (Boeckmann et al., 2003), COG (Tatusov et al., 2000) and KEGG (Kanehisa and Goto, 2000) databases. Unigenes were then aligned using BLASTN (e-value < 10^−5^) to the NCBI non-redundant nucleotide (nt) nucleic acid database, and proteins with the highest sequence similarity to the given unigenes were retrieved along with their protein functional annotations. Homologous genes in *A. crassicarpa* were identified using a BLASTp search of putative protein sequences, with an e-value threshold of <1e–10 and > 90% identity. Annotation of the unigenes with GO terms was performed using the Blast2GO software based on BLASTX hits against the NCBI nr database (e-value < 10^−5^). The quality of the unigenes was evaluated *via* BUSCO analysis using the gVolante website (Nishimura et al., 2017), which categorizes unigenes into complete, fragmented, duplicated, and missing genes. WEGO software (Ye et al., 2018) was used to perform GO functional classification of the height of the unigenes and plot the macro-level distribution of the *Acacia* database. The regulation of gene expression by transcription factors (TF) is common and important for cellular response. To characterize the operational TF families underneath the dedifferentiating *A. crassicarpa*, 41,716 amino acid sequences deduced from the unigene sequences were subjected to transcription factor enrichment analysis in Plant TFDB search (Jin et al., 2017).

### 2.5 EST-SSR analysis for polymorphism

The MIcroSAtellite (MISA) identification tool ver. 2.0 in OmicX (Beier et al., 2017) was used to search for repeated nucleotide motifs. The tool predicted the presence of multiple sets of SSR in the assembled unigenes and the allele sizes of the amplified PCR products of the candidate genes were measured. The sequence search for SSRs was performed by setting the search parameters to identify at least five repeat units of SSR containing a maximum of 10 base pairs. The software requires the input file to be the sequence in which SSRs are to be found in FASTA format, and the output file contains the name of the sequence in which the SSR is detected, the repeat motif of the SSR, the number of repetitions, the start and end of the repeat, and the length of the sequence. The number, frequency, and distribution of repeats in the SSR motifs were recorded.

To obtain experimental evidence, pairs of primers were designed for the region where the SSR sequences were predicted with an intended product size of approximately 50-bp. Fluorescently labelled PCR primers were prepared using U-19 universal reverse primers (5’-gttttcccagtcacgacgt-3’) labelled with four fluorescent molecules, 6-FAM, VIC, NED, and PET. Each labelled U-19 primer was tagged with a 2-bp barcode at the 3’ end: TG for 6-FAM, AC for VIC, CA for NED, and GT for PET. Non-labelled forward primers had a pigtail (5’-gtttctt-3’) at the 5’ end. The PCR mixture was prepared at a volume of 20 µL using the Type-it Microsatellite PCR Kit (Qiagen, Germany) containing 50–100 ng of template DNA, 0.2 µM of each labelled U-19 primer, 0.2 µM of each pigtailed forward primer and 0.04 µM of each U-19-fused reverse primer. The PCR conditions were as follows: 95°C for 5 min, followed by 10 cycles of 95°C for 30 s, 58°C for 90 s with a decrease of 0.5°C in each cycle, and 72°C for 30 s, followed by 25 cycles of 95°C for 30 s, 53°C for 90 s, and 72°C for 30 s. The final extension step was performed at 60°C for 30 min. Fragment analysis was conducted on an ABI 3130xl Genetic Analyzer (Applied Biosystems) using a LIZ-600 size standard (Applied Biosystems). Allele sizes were scored using GeneMapper 4.1 (Applied Biosystems, Foster City, CA).

## 3 Results

### 3.1 Paired-end illumina sequencing and *de novo* assembly

Total RNA was extracted from cultured cells and mRNA was fractionated using poly T-tailed affinity beads with an automated Maxwell RSC48 System (Promega). Fractionated mRNA (59.5 μg/mL, 0.2 mL) was obtained, and cDNA was prepared and subjected to Illumina HiSeq 2000 sequencing. The raw data comprised 124,410,892 reads containing 12,565,500,092 bp, which were filtered to remove cloudy reads, low-quality reads with ambiguous N bases, reads in which >10% of the bases had Q < 20, and flanking adapter sequences (**Table 1**). Clean reads were assembled to recover full-length transcripts across a wide range of expression levels (Grabherr et al., 2011). After stringent quality assessment and data filtering, 123,829,386 clean reads were obtained with a total of 12,473,930,687 bases. The Q20 was 97.64%, and the guanine–cytosine (GC) content was 45.9%. Using Trinity, all clean reads were assembled de novo into 93,317 unigenes, with a total of 84,411,793 bases and an N50 length of 1,563 bp. The average unigene length 905 bp was comparable to that reported by recent studies on avocado, Persea americana (average unigene length, 988 bp; N50 = 1,050) (Vergara-Pulgar et al., 2019); raspberry, Rubus idaeus ‘Heritage’ (average, 1,168 bp; N50 = 2,046) (Travisany et al., 2019); and Salvia guaranitica L. (average, 1,039 bp; N50 = 1,603) (Ali et al., 2018).

**Table 1:** Summary of RNA-seq Analysis of *A. crassicarpa*.

Items	Number	
Illumina HiSeq 2000 sequencing		
Total raw reads	124,410,892	read
Total raw nucleotide (nt	12,565,500,092	base
*De novo* Gene Assembling by Trinity		
Total clean reads	123,829,386	read
Total clean nucleotide (nt)	12,473,930,687	base
Q20%^1^	97.64	%
GC^2^ percentage	45.9	%
Gene Ontology		
Total unigenes	93,317	read
Total clean nucleotides (nt)	84,411,793	Base in total
N50 length	1,563	bp
Average unigene length (nt)	905	bp
Total annotated Unigenes	46,788	(50.14%)
EST-SSR search by MIcroSAtellite		
Total number of SSR-containing sequences	18,141	
Number of plural SSRs in a single contig^3^	3,875	
Number of tetramer-nucleotide-SSR	135	
Number of pentamer-nucleotide-SSR	14	
Number of hexamer-nucleotide-SSR	14	

^1^Q20% is the percentage of nucleotides with a quality value greater than 20.

^2^GC, guanine–cytosine.

^3^The number of nucleotides in a repeated unit is 4 for tertramer-, 5 for pentamer-, and 6 for hexamer-nucleotide-SS.

### 3.2 Quality assessment of assembled unigenes by benchmark universal single-copy ortholog analysis

The assembled unigenes were subjected to BUSCO analysis using the gVolante website (Nishimura et al., 2017) for a completeness assessment. BUSCO performs a like-for-like comparison, which allows categorization of assembled unigenes into complete, fragmented, duplicated, or missing genes. About 59% (853) of the unigenes were categorized as ‘complete,’ 23% (334) were ‘duplicate,’ 5% (68) were ‘fragmented,’ and 13% (185) were missing from the A. thaliana reference.

The term ‘complete’ refers to a unigene within two standard deviations (2σ) of the BUSCO group’s mean aligned sequence length. Unigenes outside of this limit were classified as ‘fragmented’ transcripts. The ‘duplicated’ category refers to unigenes that match multiple BUSCO reference genes, fulfilling both the ‘expected-score’ and the ‘expected-length’ cut-offs. ‘Missing’ unigenes are the ones that do not meet the expected score cut-off. The low percentage of fragmented (5%) and missing (13%) unigenes substantiated the quality of the assembled unigenes.

**Figure 1:**
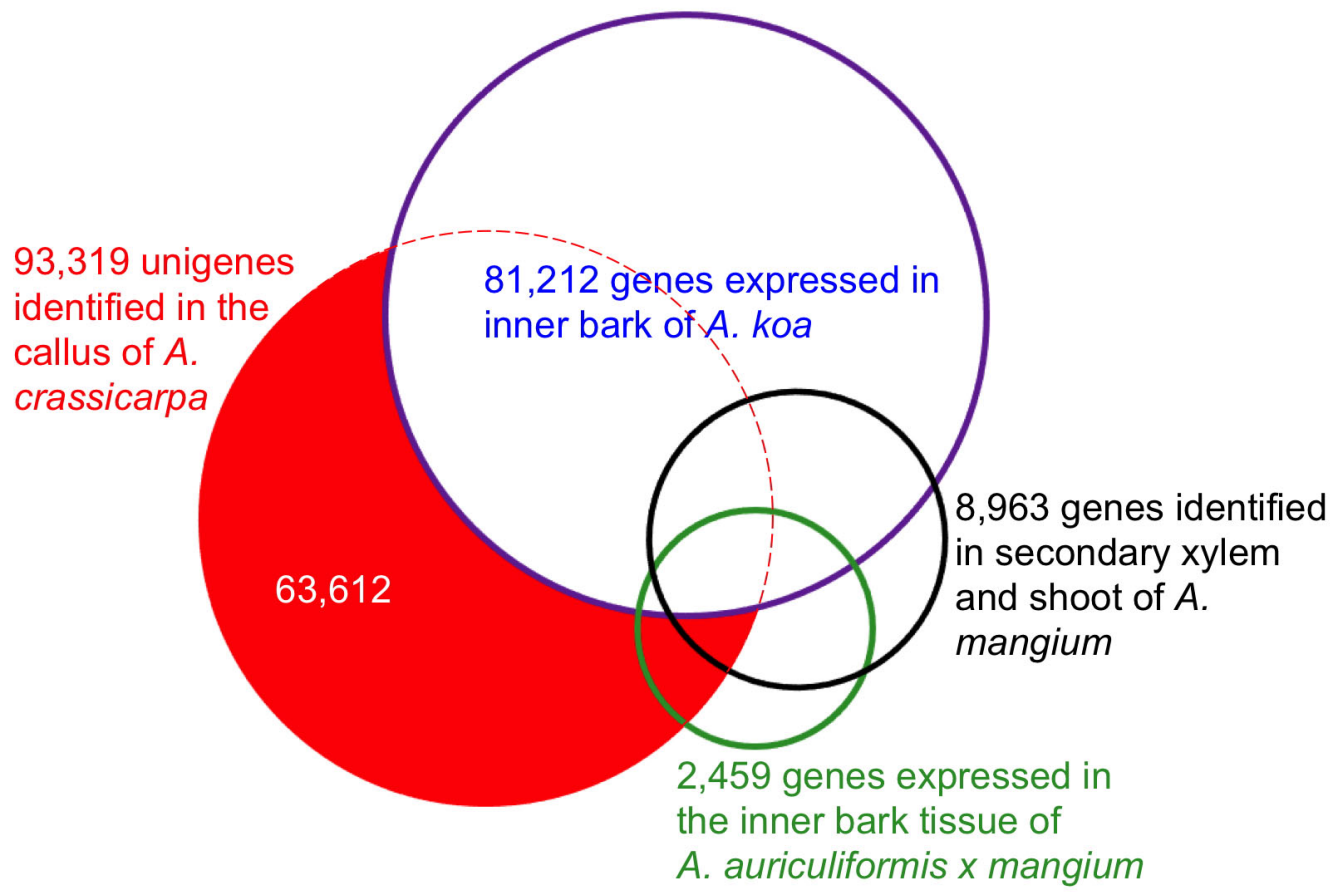
Overlap expressed genes in the callus of *A. crassicarpa* compared to the previously published EST database. Putative orthologous genes were defined by blastn search with the e-value cutoff 1xe^-10.

To further investigate the relatedness of the assembled unigenes to closely related tree species, differentially expressed genes were mapped to Acacia ESTs using all-versus-all pairwise comparison. The 93,317 unigenes identified in the present study were aligned against 81,212 genes expressed in the inner bark of Acacia koa (Lawson and Ebrahimi, 2018), 8,963 genes expressed in the secondary xylem and shoot of Acacia mangium (Suzuki et al., 2011), and 2,459 ESTs in the bark of Acacia auriculiformis × A. mangium (Yong et al., 2011). The similarities are shown in the Venn diagram (**Figure 1**). There were 29,707 overlapping unigenes between A. crassicarpa and the inner bark of A. koa, accounting for 44% and 37% of the assembled unigenes, respectively. The proportion of overlapping unigenes between A. crassicarpa and the secondary xylem and shoot of A. mangium was only 9%, representing a smaller library comprising 8,963 unigenes. The ESTs identified in the inner bark tissues of Acacia auriculiformis × mangium comprised a small number of 2,459 genes, and the A. crassicarpa unigenes identified in our transcriptomic library shared only 7% gene identity overlap. This indicates that the number of genes that next-generation sequencing can decode is an order of magnitude higher than the number of genes that EST sequencing can decode.

### 3.3 Functional annotation and GO-term assignment

**Figure 2:**
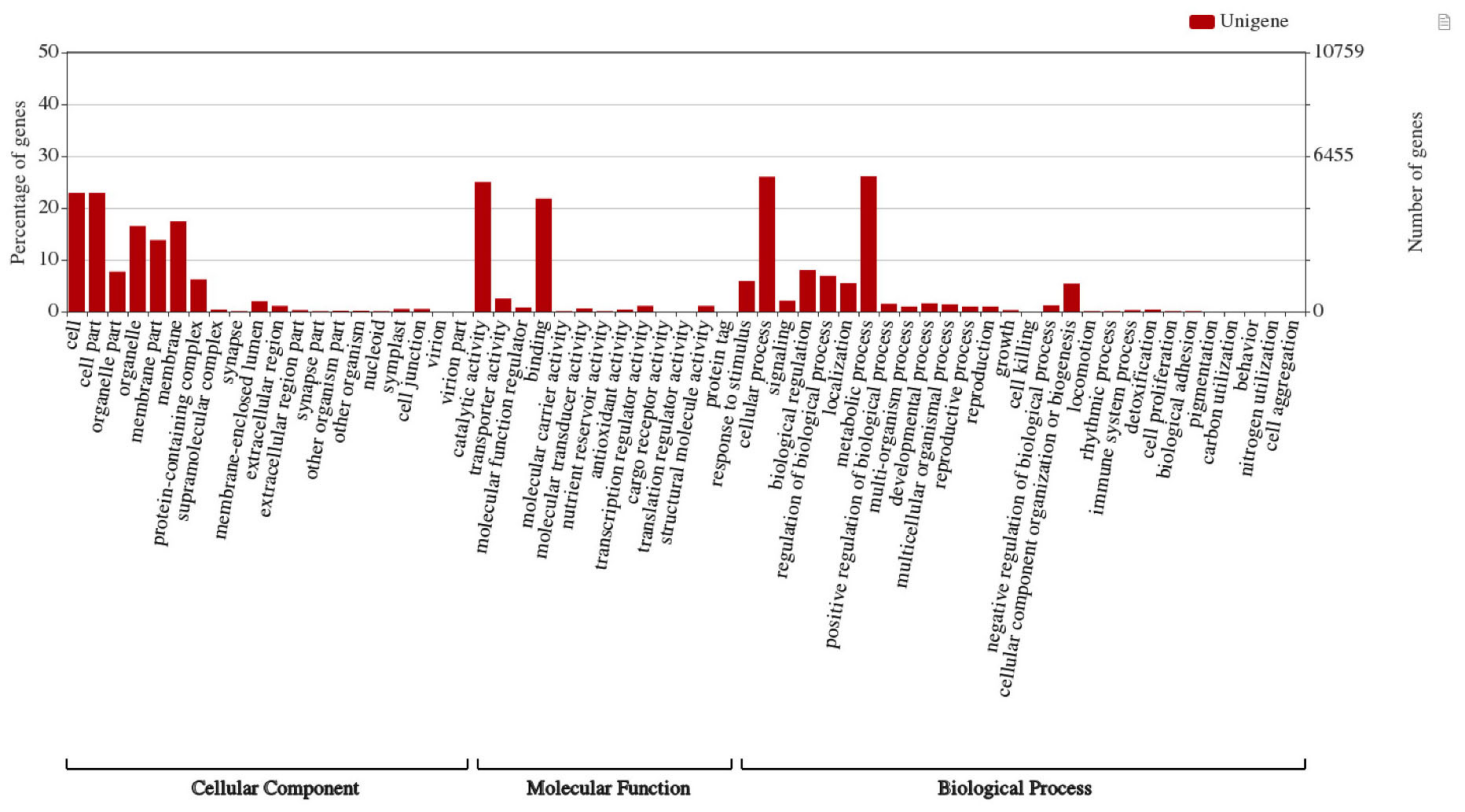
Gene ontology categories of assembled unigenes in *A. crassicarpa* callus shown by WEGO web application. GO terms were assigned in three processes regarding cellular component, molecular function and biological process.

The Blast2GO program (Conesa et al., 2005) was used to annotate the assembled 93,317 unigenes, and Gene ontology (GO) terms were assigned to 46,788 (50.14%) unigenes in reference to the NCBI nr, NCBI nt, Swiss-Prot, Clusters of Orthologous Groups (COG), and Kyoto Encyclopedia of Genes and Genomes (KEGG) databases for annotation and validation. Base alignment with an e-value of < 10-5 was selected. To perform the GO functional classifications for all the ‘complete’ unigenes, the WEGO software (Ye et al., 2018) was used to examine the macro-level distribution of gene functions for this species. Of the complete unigenes assembled in our study, GO terms assigned by BLAST2GO were used in WEGO to categorize GO functional classifications. The Unigenes were categorized into three major categories: cellular components (31%), molecular functions (36%), and biological processes (33%). The Unigenes were assigned to cellular components (22.9%), cell parts (22.9%), membranes (17.4%), membrane parts (13.8%), and organelles (16.5%) (**Figure 2**). Molecular functions of the unigenes included catalytic activity (25%) and binding (21.8%). The biological processes of the unigenes included cellular (26.0%) and metabolic (26.1%) processes.

### 3.4 Metabolic pathway reconstruction by KEGG mapping

**Figure 3:**
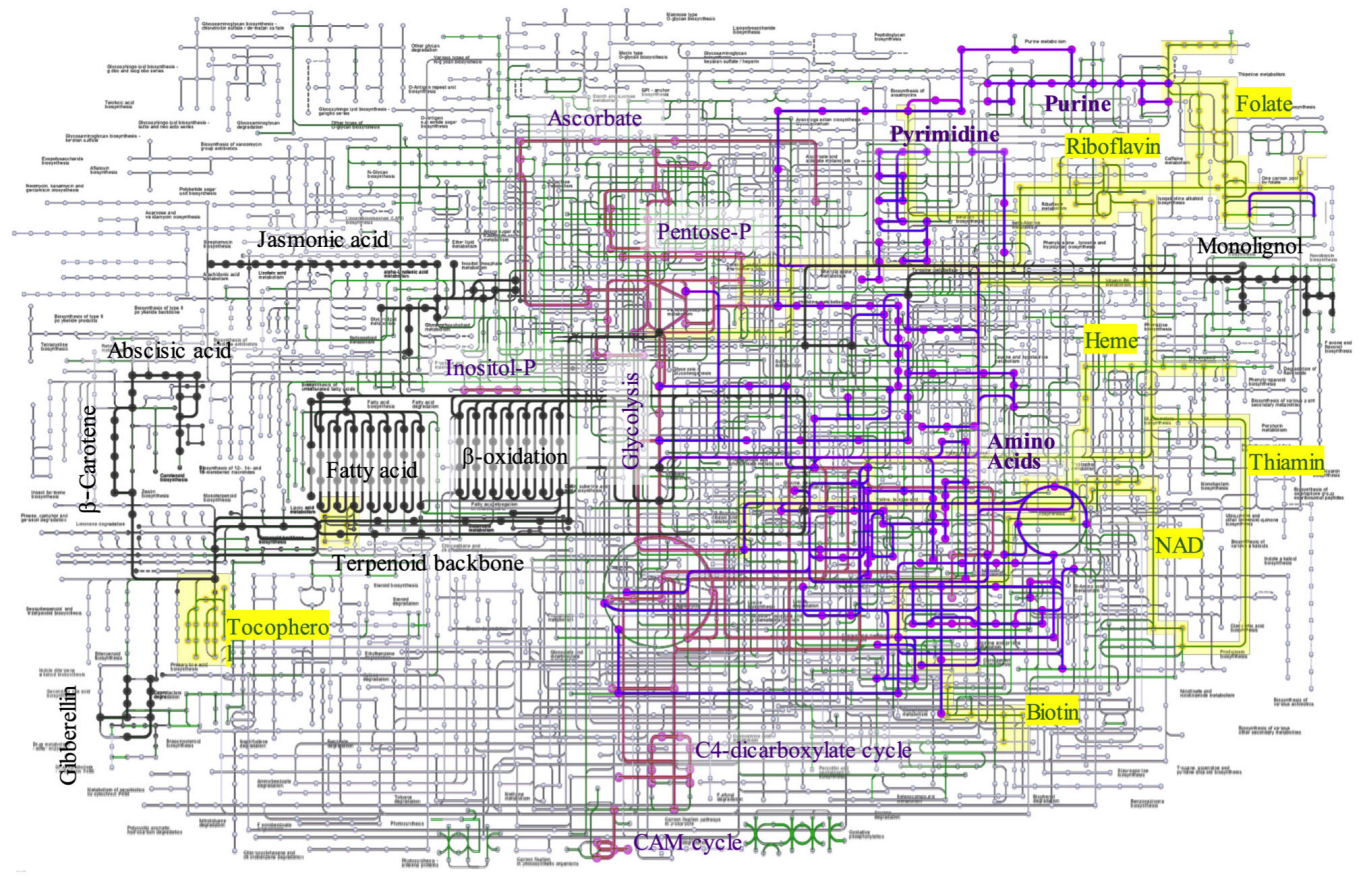
Metabolic pathway construction by KEGG mapping. The unigenes annotated to metabolic enzymes were reconstructed to KEGG metabolic map. Carbohydrate and Energy metabolism (pink), Nucleoside and amino acid metabolites (purple), lipid, terpen, and lignin (black), cofactors (yellow), and others (green).

The KEGG database was used to systematically assign annotated unigenes to the metabolic pathways. KEGG mapping successfully categorized 16,662 unigenes, which accounted for 35.6% of the total unigenes, in accordance with the hierarchal annotation system KEGG BRITE, into the three major protein families involved in metabolism (2,406 unigenes), genetic information processing (2,666 unigenes), and signaling and cellular process (595 unigenes). The metabolic pathways were reconstructed by mapping the functionally annotated unigenes onto pathways for carbohydrate and energy metabolism, nucleotide and amino acid metabolism, lipids, terpenes, lignin metabolism, and cofactor/vitamin biosynthesis (**Figure 3**). The number of genes involved in metabolic pathways was the largest for carbohydrate and energy metabolism, accounting for 666 unigenes in 35 pathways, followed by biosynthetic pathways for amino acids and nucleotides, which involved 440 unigenes in 23 pathways. The metabolic pathways for lipid, terpenoid, and lignin metabolism accounted for 277 genes in the 27 pathways. Biosynthesis of cofactors and vitamins accounted for 179 unigenes in the 12 pathways.

Several important genes were involved in the monolignol biogenesis pathway, including phenylalanine/tyrosine ammonia-lyase [EC:4.3.1.25], trans-cinnamate 4-monooxygenase [EC:1.14.14.91], 4-coumarate-CoA ligase [EC:6.2.1.12], shikimate O-hydroxycinnamoyltransferase [EC:2.3.1.133], 5-O-(4-coumaroyl)-D-quinate 3’-monooxygenase [EC:1.14.14.96], caffeoyl-CoA O-methyltransferase [EC:2.1.1.104], cinnamoyl-CoA reductase [EC:1.2.1.44], ferulate-5-hydroxylase [EC:1.14.-.-], and caffeic acid 3-O-methyltransferase/acetylserotonin O-methyltransferase [EC:2.1.1.68 2.1.1.4]. The monolignol biosynthetic pathway is well characterized, but the coordination and regulation of cell wall formation are not well understood. Several transcription factors may be involved in the formation of lignin and wood formation (Zhang et al., 2021).

### 3.5 Identification of putative transcription factors

Plant Transcription Factor Database (PlantTFDB 4.0) predicted 756 transcription factors that matched the homologues of A. thaliana (E-value < 10-5) (**Table 2**) and classified them into 40 families. The most abundant family was the bHLH13-like protein (78 unigenes), followed by the ethylene-responsive element (ERF) (62 unigenes), MYB family (55 unigenes), WRKY DNA-binding proteins (54 unigenes), bZIP family (53 unigenes), and lysine-specific histone demethylase (53 unigenes), as well as others that were lower in number. It has been reported that major transcription factors, ERF (Ikeuchi et al., 2022), MYB (Sakamoto et al., 2022), bHLH and WRKY (Xu et al., 2023a), play pivotal roles in reprogramming in response to physical injury and hormone signaling.

**Table 2:** Annotation of putative transcription factors.

Description	Unigenes
Transcription factor bHLH13-like	78
Ethylene Responsive Element	62
MYB family protein	55
WRKY DNA-binding protein	54
bZIP family protein	53
Lysine-specific histone demethylase (C2H2)	53
MYB-related family protein	48
CCCH-type zinc finger protein	33
Trihelix	33
FAR1 family protein	30
GRAS family protein	30
G2-like Family Protein	28
Nuclear Factor Y	26
Prosopis alba B3 domain-containing protein	19
AP2-like ethylene-responsive transcription factor	16
DOF family protein	15
Homeodomain-like transcriptional regulator	13
GATA transcription factor	12
M-type_MADS	12
TCP Family Protein	10
BEACH domain-containing protein B	8
SBP family protein	7
Prosopis alba two-component response regulator ARR2-like	6
DBB family protein	6
DNA-binding storekeeper protein-related transcriptional regulator	6
Prosopis alba protein BASIC PENTACYSTEINE6-like	5
CO-like	5
DP-E2F-like 1	5
CPP	4
BES1/BZR1 homolog protein 2	3
CAMTA	3
LSD family protein	3
VOZ protein	3
WOX Family protein	3
Ethylene-Insensitive Protein	2
Pathogenesis-related homeodomain protein-like	2
RAV Family Protein	2
Prosopis alba growth-regulating factor 3-like	1
Prosopis alba protein Effector of Transcription 2-like	1
ZF-HD	1
	756

### 3.6 Unigenes involved in phytohormone signaling

Acacia crassicarpa expressed genes involved in all known plant hormone signaling pathways (**Figure 3**). Unigenes encoding components of auxin (AUX), gibberellin (GA), and jasmonic acid (JA) signaling were annotated. Most of the unigenes involved in cytokinin (CK) and abscisic acid (ABA) signaling were also annotated, except for the B-ARR transcription factor in CK signaling, and EIN2 and BSU1 were involved in phosphor-relaying components. Signaling components for ethylene (ET), brassinosteroids (BR), and salicylic acid (SA) were all expressed in the undifferentiated cells of A. crassicarpa.

### 3.7 SSR analysis for polymorphism

**Figure 4:**
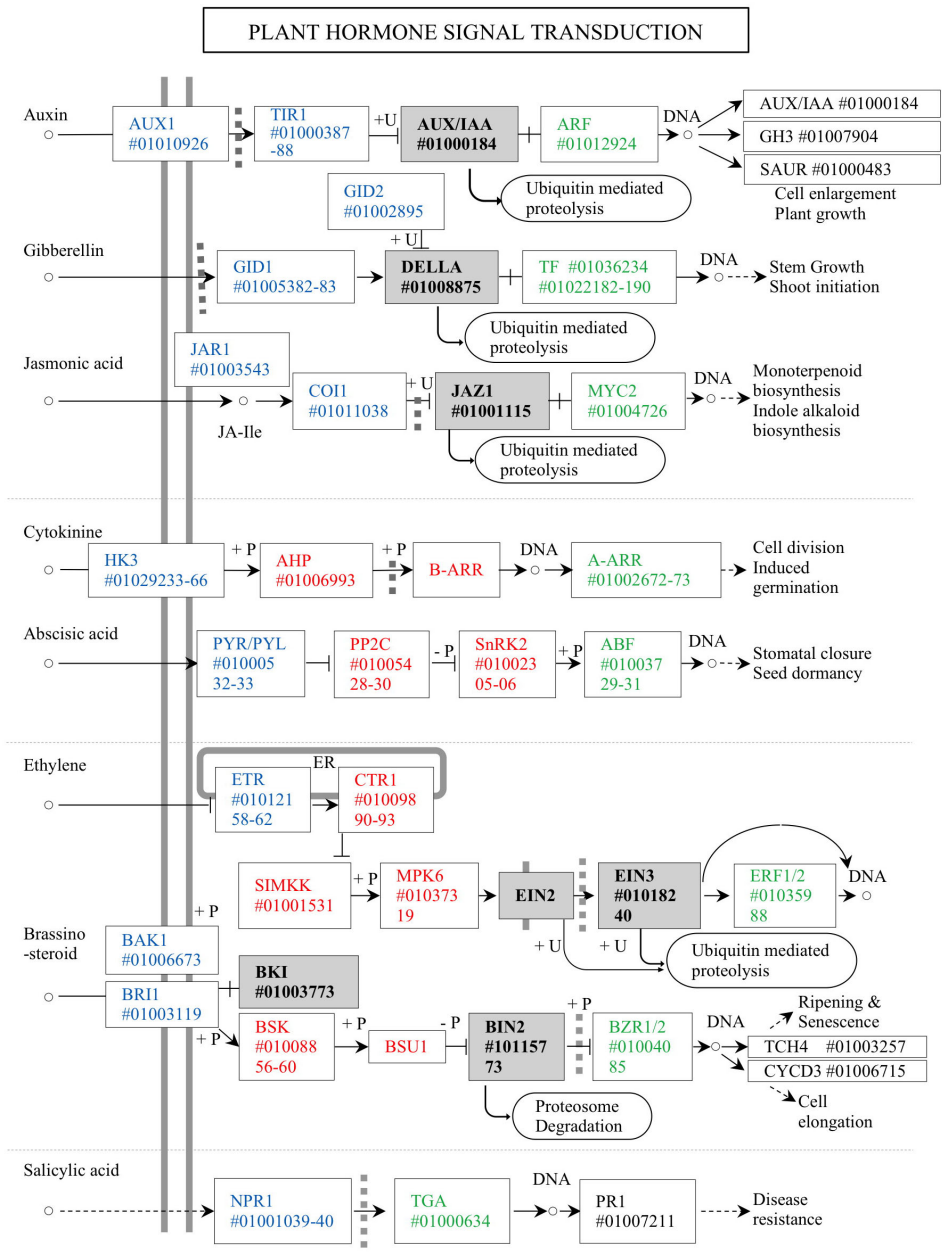
Unigenes annotated for the plant hormone signaling in the *A. crassicarpa* transcriptome. Signal transduction components are classified as ligand receptors (blue), Phospho-relay components (red), transcription factor regulators (gray), and transcription factors (green). The unigene identities are specified as ICSL numbers starting with a # symbol.

The assembled unigene sequences of A. crassicarpa were subjected to MISA to identify SSR representing di-, tri-, and tetra-nucleotide repeats. Dinucleotide SSRs (AG, AT, and AC) were more frequent than trinucleotide SSRs (**Figure 4**). Among the trinucleotide repeat motifs, the dominant repeat motif was (AAG)n, followed by (AGG)n, (AAT)n, (ATC)n, and the other trinucleotide repeats. Dinucleotide repeats occur more frequently than tri- or tetranucleotide repeat units. Among the dinucleotide repeat motifs, the most frequent repeat detected in this study was AG/CT, followed by AT/TA, and AC/GT. The least frequent dinucleotide was GC/CG. This finding is consistent with those of previous studies conducted on Stevia rebaudiana (Kaur et al., 2015), the traditional Chinese medicinal plant Epimedium sagittatum (Zeng et al., 2010) and coffee (Aggarwal et al., 2007).

Primers were designed to detect polymorphisms by targeting unigenes that contained triplet repeats in their ORFs (**Table 3**). Among the 16 DNA samples from local A. crassicarpa trees, three multiple signals of 258, 287, and 290-bp were observed for the SSR marker designed for the unigene ICSL01027786 (293bp), which was annotated as auxin response factor 3 (ARF3). Three signals were also produced from the unigene ICSL01000311 (266 bp), which was annotated as ARF4 (**Table 4**). Other unigenes annotated as transcription factors, such as ICSL01001643 (254bp), ICSL01023085 (175 bp), and ICSL01008756 (216 bp), also produced multiplicity in SSR analysis. Unigene ICSL01001643, annotated as a putative ethylene-responsive transcription factor (Charfeddine et al., 2019), produced two PCR bands with lengths of 214 and 277 bp. Unigene ICSL01023085 encodes a proline-rich PRCC (Weterman et al., 2000) and exhibits polymorphism with four PCR bands of 197, 200, 249, and 257 bp. Unigene ICSL01008756 was annotated as tubby-like F-box protein 5 (Song et al., 2019) (Xu et al., 2016);, and its polymorphisms appeared as PCR products with lengths of 235, 238, and 241 bp. Moderate multiplicity giving signals of 179 and 182-bp was observed for unigene ICSL01034178 (154 bp), which was annotated as a pollen tube guidance gene (Li et al., 2015). The polymorphic information content (PIC), which represents the ability to detect polymorphisms, was calculated using Cervus (Kalinowski, 2007), and was high (PIC > 0.4) for ARF3, ARF4, PRCC, and Tubby-like F-box protein 5, and moderate (PIC < 0.4) for ERTF and PTG.

**Table 3:** PCR primers for SSR marker amplification.

Target gene ID		Primers
c26532_gl_il	F	gtttcttACGATGAAACACCGTCGTCT
	R	gttttcccagtcacgacgt CA CGACAGAACACGTGAAATGG
c11193_gl_i1	F	gtttcttCCCAGCACACATTCAGAAGA
	R	gttttcccagtcacgacgt GI CAAAGCACAAGACGTGAAGO
c13147_g2_i1	F	gtttctTCTTATTCAACCCCTGAGGC
	R	gttttcccagtcacgacgt CA GAGAAAGACATTGACGGGGA
c25518_g1_i1	F	gtttctTCCCTCCCCAAACCTAAATC
	R	gttttcccagtcacgacgt TG CGTTTTCTGCTTTACGCTCC
c20030_g1_i1	F	gtttcTTGGATAGTCAGCTTCCGCT
	R	gttttcccagtcacgacgt CA CCCTTCTTGGATGGCAGATA
c27691_gl_i1	F	gtttcttGCCTTGCTTCGATCTTGTTC
	R	gttttcccagtcacgacgt GT ACTTGGATCGGCTAAAGGGT

The forward primer (F) has a pigtail motif (5’-gtttctt-3’) at the 5’ end. The reverse primer (R) has a U-19 universal reverse sequence (5’-gttttcccagtcacgacgt-3’) that is labelled with four kinds of fluorescent molecules (6-FAM, VIC, NED, and PET). The Labelled U-19 primer was also tagged with 2 bp barcodes at the 3′ end (TG for 6-FAM, AC for VIC, CA for NED, and GT for PET).

**Table 4:** Polymorphism represented as the length distribution of PCR products focused on SSR regions.

Contig	ICSL01027786			ICSL01000311			ICSL01001643		ICSL01023085				ICSL01008756			ICSL01034178	
Repeat	CTT x 5			CAT x 5			TGC x 5		GAC x 6				GCT x 5			CTT x 5	
Annotation	ARF3			ARF4			ERTF		Pro-rich PRCC				Tubby-like F-box protein 5			PTG	
Tree 01	258	287			286	292	214	277	197		249		235	238		179	
Tree 02	258		290			292	214	277	197		249			238			182
Tree 03		287				292		277	197			257		238		179	182
Tree 04	258	287		280		292	214	277		200	249			238		179	182
Tree 05	258	287			286	292	214	277	197		249			238			182
Tree 06	258		290			292	214	277	197		249			238			182
Tree 07	258	287		280	286		214	277			249	257	235		241	179	
Tree 08	258	287			286	292	214	277	197		249		235	238		179	
Tree 09	258	287			286	292	214	277		200	249			238		179	182
Tree 10	258		290		286	292	214	277	197		249		235	238		179	182
Tree 11	258	287			286	292	214	277	197		249			238		179	
Tree 12	258	287	290			292	214		197		249		235			179	
Tree 13	258		290			292	214	277		200	249		235		241	179	
Tree 14	258	287	290		286	292	214	277	197		249			238		179	
Tree 15	258	287	290	280	286		214	277		200	249			238		179	182
Tree 16	258	287			286	292	214	277		200	249		235		241		182
H_o_	0.938			0.688			0.938		1				0375			0.313	
He	0.655			0.558			0.514		0.675				0.514			0.498	
PIC	0.561			0.464			0.374		0.593				0.436			0.366	
F(Null)	-0.223			-0.1104			-0.306		-0.2309				0.1934			0.2138	
Position	CDS			CDS			CDS		CDS				CDS			CDS	

The microsatellite locus was amplified by PCR using a primer pair flanking the in-frame SSR region. Polymorphisms were observed in contigs encoding auxin response factors (ARFs), ethylene response transcription factor (ERTF), and pollen tube guidance protein (PTG). The length of the PCR products presented in the table was measured by size calibration using the LIZ-600 standard marker. Ho, observed heterozygosity; He, expected heterozygosity; PIC, polymorphic information content; F (null), estimated null allele frequency; CDS, Coding sequence for protein.

## 4 Discussion

### 4.1 Informative foundation for the molecular breeding of *A. crassicarpa*

Molecular genetics provides valuable information for developing programs for efficient tree breeding. Several commercially important traits of trees, such as fast growth, few branches, high strength, and resistance to pathogens, are likely to be controlled by multiple layers of gene regulation. Although these traits are difficult to control by environmental management such as optimized fertilization, several genes are involved in the commercially important traits of various plants. The present study reports a transcriptomic analysis utilizing next-generation sequencing, which offers an effective approach to genomic resources at a reasonable cost in a relatively short period, even for non-model tree species.

### 4.2 Annotation of unigenes for metabolic enzymes and transcription factors

Annotation of genes encoding metabolic enzymes and mapping on the KEGG metabolic pathway provides an atlas that describes the metabolic flow in the pluripotent cells of A. crassicarpa. Potential targets, such as those involved in monolignol biosynthesis, were among the genes involved in the primary metabolic pathways. This genetic information may offer a candidate for selective breeding and genome editing programs to increase the pulp yield and reduce pulping costs. Annotation of transcription factors also provides another set of candidates to be disrupted by genome-editing technology, thereby modifying the texture of lignin content and hormone responses, including pathogen resistance.

The cellular reprogramming process involved several genetic perturbations induced by physical injury of explant (Deng et al., 2022) (Park et al., 2023b), and supplementation of auxin and cytokinin (Asghar et al., 2023). Elucidation of the gene regulatory mechanism, involving several transcription factors (Kumar et al., 2021, Dai et al., 2020, Xu et al., 2023b), signaling pathway components (Park et al., 2023a), and epigenetic alteration of chromatin (Chen et al., 2023) may give us clue how to reverse the transformed callus back to the differentiated plant body.

### 4.3 Plant hormone signaling as the genetic switch

Phytohormones can provide an effective means of operating genetic switches from outside the hard bark with reproducible and quantitative responses. We considered the nature of these switches in terms of their reversibility, dose responsiveness, and mechanisms that enable crosstalk with other hormones. The three phytohormones auxin (AUX), gibberellin (GA), and jasmonic acid (JA) exploit ubiquitin-dependent proteolysis, which eliminates the specific regulator proteins that negatively control transcription factors. The elimination of negative regulators allows transcription factors to activate hormone-responsive gene expression. The switchover to the signal-responding state continues until lifted restrictions are regenerated through the biosynthesis of negative regulators. Therefore, hormone regulation via AUX, GA, and JA could provide a binary on/off switch.

Cytokinin (CK) and abscisic acid (ABA) signaling is transmitted through a phosphorylation relay between receptors and regulators. Phosphorelay-dependent regulation involves a series of phosphorylation events that lead to transcription factor activation. Phosphorylation, typically catalyzed by protein kinases, can occur in multiple steps, creating a phosphorylation gradient. The extent of phosphorylation determines the activity level of the transcription factor, and consequently, the strength of the signaling response. Accordingly, this gradient regulation allows fine-tuning of the response, enabling different levels of gene expression based on the concentration or duration of the hormone signal.

Ethylene (ET) and brassinosteroid (BR) signaling constitute the phosphorylation relay upstream; however, proteolytic removal of the transcriptional regulator is involved downstream. Such a hybrid mechanism can allow a quantitative response to intermediate signaling components, which may mediate crosstalk with other hormone signaling (Yasumura et al., 2015; Wang et al., 2020; Zhao et al., 2023), while the proteolytic switchover to gene expression provides a determinative response to hormone stimuli by the accumulated signaling of ET (Chen et al., 2022) or that of BR (Kour et al., 2021). In the systemic immune response induced by salicylic acid, the response to the SA receptor NPR1, which undergoes transformation from an oligomeric state in the cytosol to monomers upon binding to SA, activates pathogenesis-related genes in the nucleus (Palmer et al., 2019). The straightforward transmission of SA signaling allows a quick response to pathogen invasion.

### 4.4 SSR marker development

Individual trees may exhibit slight but discernible differences in growth rate, lignin content, stress tolerance, and pest resistance. The genetic traits underlying these characteristics may be associated with molecular genetic markers, which provides a scientific foundation for describing these tree characteristics. Short sequence repeats (SSR) are nucleotide regions that represent tandem repeat units located in coding and non-coding regions of the genome (Gupta et al., 1996).

None of the 16 subspecies of A. crassicarpa was identical in terms of the PCR products amplified by pairs of primers flanking the repeated trinucleotide sequences. The highest similarity in the EST-SSR products was observed between subspecies 05 and 11, which differed only in the PCR products from unigene ICSL01034178, which was annotated as a pollen tube guidance protein. The highest difference in polymorphism was observed between subspecies 03 and 04, which exhibited diverse multiplicities, except for ICSL01008756 and ICSL01034178. Polymorphisms in these transcription genes may exert a significant effect on the set of genes expressed under these regulators, which may be relevant to the physiological diversity of the 16 subspecies of A. crassicarpa.

**Figure 5:**
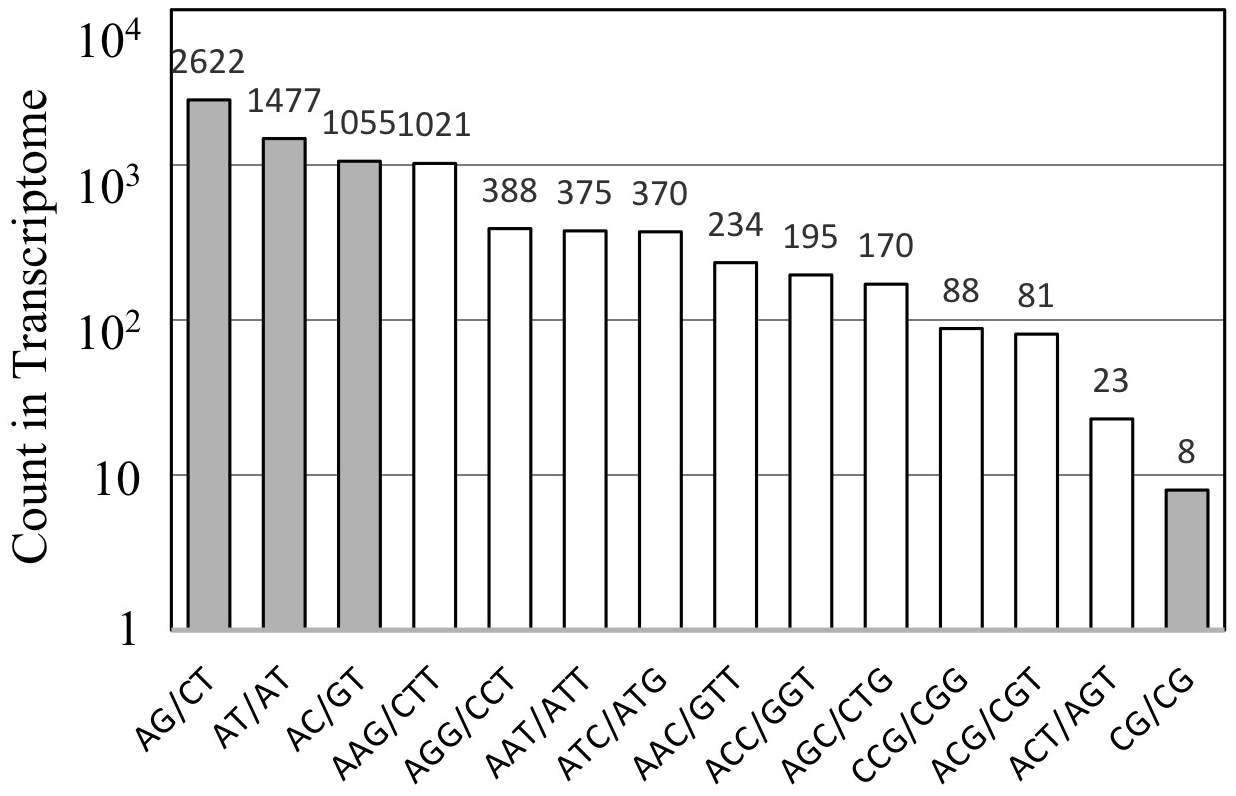
The distribution of SSRs identified from the *A. crassicarpa* transcriptome. Occurrence of repeat sequences with di- and tri-nucleotide units are designated in gray and white, respectively.

Although the predominant trinucleotide repeats contained adenine-leading motifs such as AAG, AGG, AAT, and ATC (**Figure 5**), polymorphisms were produced when targeting repeats started with G or C, such as CTT, CAT, and GCA (**Table 4**). The high occurrence of repeated units does not necessarily guarantee successful targeting by SSR fingerprinting.

SSR fingerprinting can be produced by PCR amplification of SSR on assembled transcriptomic sequences (transcriptome-SSRs), ESTs (EST-SSRs), or genomic DNA sequences (genomic SSRs). Transcriptome SSR sequences are more accessible than genomic SSR markers because they can focus on gene-rich regions and are associated with transcription. Transcriptome-SSR markers contribute to the segregation of closely related subspecies and variants, and these markers may be used in breeding programs to improve the quality and biomass of trees as a fiber resource and to achieve the desired properties, such as the ability to lower the salt content in combustion ash when used as biomass fuel (Thiel et al., 2003; Chung et al., 2014).

In our future studies, gene co-expression networks may be constructed using time-resolved transcriptomic approaches as illustrated for maize (Du et al., 2019) soybean (Park et al., 2023a) and cotton (Fan et al., 2022), which will enable the clustering of gene expression at each phase of the cellular reprogramming process from the differentiated plant body to the pluripotent state. The molecular genetic sequence information established in the present study will assist in the molecular breeding of *Acacia* trees with SSR markers and functional annotation of genes and assignment to metabolic map pathways.

## Data Availability

The datasets presented in this study can be found in online repositories. The names of the repository/repositories and accession number(s) can be found below: https://www.ddbj.nig.ac.jp/, SAMD00213802, https://www.ddbj.nig.ac.jp/, PRJDB9506, https://www.ddbj.nig.ac.jp/, DRX208625, https://www.ddbj.nig.ac.jp/, DRR218312.
